# A Powerful Statistical Approach for Large-Scale Differential Transcription Analysis

**DOI:** 10.1371/journal.pone.0123658

**Published:** 2015-04-20

**Authors:** Yuan-De Tan, Anita M. Chandler, Arindam Chaudhury, Joel R. Neilson

**Affiliations:** Department of Molecular Physiology and Biophysics and Dan L. Duncan Cancer Center, Baylor College of Medicine, Houston, Texas, 77030, United States of America; Indiana University Bloomington, UNITED STATES

## Abstract

Next generation sequencing (NGS) is increasingly being used for transcriptome-wide analysis of differential gene expression. The NGS data are multidimensional count data. Therefore, most of the statistical methods developed well for microarray data analysis are not applicable to transcriptomic data. For this reason, a variety of new statistical methods based on count data of transcript reads have been correspondingly proposed. But due to high cost and limitation of biological resources, current NGS data are still generated from a few replicate libraries. Some of these existing methods do not always have desirable performances on count data. We here developed a very powerful and robust statistical method based on beta and binomial distributions. Our method (mBeta t-test) is specifically applicable to sequence count data from small samples. Both simulated and real transcriptomic data showed mBeta t-test significantly outperformed the existing top statistical methods chosen in all 12 given scenarios and performed with high efficiency and high stability. The differentially expressed genes found by our method from real transcriptomic data were validated by qPCR experiments. Our method shows high power in finding truly differential expression, conservatively estimating FDR and high stability in RNA sequence count data derived from small samples. Our method can also be extended to genome-wide detection of differential splicing events.

## Introduction

The optimization of next generation sequencing (NGS) technologies in recent years [1,2,3] has led sequencing cost to rapidly decline so that the sequencing technologies as platforms for studying gene or sub-gene expression have become more and more attractive[4,5,6]. Current NGS technologies such as Tag-seq [7], deepSAGE [8], SAGE-seq [9], and PAS-seq [10] can generate short reads of sequences, that is, sequences of 35–300 bp that correspond to fragments of the original RNA. In particular, 3P-seq or PAS-seq[10], a deep sequencing-based method for quantitative and global analysis of RNA polyadenylation has been broadly used to study expression behavior of RNA isoforms in a variety of human and mouse cells.

To evaluate differential expression between conditions or cases, sequences need to be mapped to a reference genome and annotated. After doing so, the sequence data can be transformed to read count data at the genomic level of interest. Although RNA-Seq technologies can be used to study differential transcription of exons, splice-variants, isoforms-specific [11,12,13,14] and allele-specific expression[15,16], our focus here is on differential expression of genes or mRNA isoforms due to differential splicing or alterative polyadenylation signals and cleavage sites in 3’ untranslated regions (3’UTR).

A NGS library may be thought of as a population of sequences and each sequence as an individual of this population. Sequencing an NGS library is a random process of sampling from this population. If each individual sequence tag has an equal chance to be selected for sequencing, then the probability of sequencing a RNA sequence will be proportional to the length of waiting time [17]. In this case, counts of reads for a given transcriptomic feature should follow the Poisson distribution, which indicates that only one parameter determines the variation of read counts. However, some read counts are over-dispersed between samples and cannot be explained by the single-parameter model. This is because the Poisson model has only one variation source. Actually, variation of many read counts derives from noise and biological effects. This is characteristic of negative binomial (NB) distribution [17,18] and binomial distribution.

To identify differential expression of RNA tags, many statistical methods have been developed so far based on normal approximation [19,20,21], permutation [22], beta distribution [23] or over-dispersed logistic/over-dispersed logistic linear distribution [24]. As RNA count data have become more and more prevalent, newer statistical methods such as edgeR Exact test [18], empirical Bayesian [25], DESeq [17], generalized linear modeling (edgeR GLM) [4], and likelihood ratio test [26] have recently been proposed.

Despite the development of technologies dramatically reduces costs of sequencing, RNA sequencing experiments are still limited to a few replicate libraries for a condition or a case. The basic need to assess differential expression within the context of biological variation remains undiminished, but this problem becomes complicated by the fact that different mRNA transcripts may have different degrees of biological variation. There is a need to estimate biological variation as reliably as possible from a limited number of replicate libraries [4]. To address this problem, existing statistical methods such as empirical Bayesian (baySeq) [25], DESeq [17] and edgeR Exact test[18] adopt variation information, that is, common dispersion, across the transcriptome. In contrast, edgeR GLM [4] uses genewise or tagwise dispersion. However, none of these methods considers a transcript-by-transcript “fudge effect” resulted from small samples. So-called fudge effect is such an effect that small samples lead to more chance of decreasing within-group variances and occurrence of gap events between groups. This is because in high-throughput data, small sample sizes would have a small chance that samples are drawn from terminals of distribution so that difference among replicates becomes small. Therefore fudge effect would results in inflation of statistics [27,28] which easily gives rise to false discoveries. The fudge phenomenon broadly exists in high-throughput data especially in transcriptomic data because there are a lot of very small counts (see Discussion Section for more detail). Suppressing such an effect can greatly improve the performance of statistical methods in identification of differentially expressed mRNA transcripts. To do so, we are required to develop novel methods based on a way different from edgeR GLM, edgeR Exact test and DESeq.

Our development work is based on Baggerly et al’s [23] work because this method is not sensitive to data distributions (see Discussion Section). Beyond this, the Beta t-test approach uses weights to exclude artificial or technical noise in count data and thus identifying differentially expressed transcripts with higher probabilities. The third, a very important point, is that the Beta t-test is a t-test method that is a classical distance-variance test approach and very clear and simple to understand differential expression of RNA isoforms but the Beta t-test is significantly susceptible to the fudge effect. For these reasons, we are highly motivated to develop a new approach specially working on count data of RNA sequences derived small samples.

## Material and Methods

### Cell lines and stimulation

Jurkat T-cell lines were obtained from the ATCC and maintained in RPMI (ATCC) with 10% fetal bovine serum supplemented with penicillin and streptomycin (Gibco). The cells were stimulated for 48 hours in plates coated with a solution of 1 mg anti-CD3 (OKT3—eBiosciences) and 5 mg anti-CD28 (CD28.2—BD Pharmingen). Activation was monitored via flow cytometric detection of CD69 expression (FN-50) 16–24 hours after stimulation.

### High-throughput sequencing library generation

Total RNA was harvested from resting and stimulated T-cells with Trizol reagent (Life Technologies) following the manufacturer’s protocol. Polyadenylated RNA was isolated with the Poly(A)-Purist MAG (Ambion/Life Technologies) kit as per manufacturer instruction. Libraries for high-throughput sequencing were constructed essentially as described [10], with the exception that “barcoded” linkers were used to facilitate multiplexing. Libraries were sequenced via 75 bp paired-end sequencing on an Illumina GAIIx in the Genomic and RNA Profiling Core at Baylor College of Medicine.

### Annotation and pipeline analyses

Sequence reads were mapped to the UCSC mm9 build of the mouse genome with bwa version 0.5.9 [29], using the paired-end mapping module, default alignment stringency, and requiring uniquely mapped proper pairs. To rescue reads crossing splice junctions, non-mapping reads were remapped to the UCSC KnownGene reference and then again projected to mm9. Mapped reads were collected into distinct polyadenylation sites (isoforms) based on the mapping coordinate of their 3' ends. Briefly, all reads mapping within a 20-nucleotide sliding window were merged, using the frequency-weighted median 3' end as the formal tag identifier. Isoforms were then filtered for false priming using a progressively filtering strategy assessing adenosine and guanine composition in the five, ten, and fifteen bases followed the isoform-mapping site. Isoform reads were counted and annotated using UCSC KnownGene annotations. For each individual transcription unit, the annotated poly(A) sizes were ranked in descending order by count. Beginning with the isoforms expressed at the highest frequency, isoforms were collected until the frequency of the collected isoforms surpassed 90% of the aggregate reads for the transcription unit. The remaining isoforms were then discarded. The counts of isoforms in all libraries were then normalized using a negative binomial model with DESeq [17] so that all replicate libraries had the same size.

### qRT-PCR

Total RNA was isolated from resting and stimulated (48 hours) Jurkat T-cells using Trizol (Invitrogen) and digested with DNase I (Invitrogen) to remove contaminating genomic DNA. One microgram of total RNA was used to template cDNA synthesis using oligod(T) SuperScript III Reverse Transcriptase. Real-time PCR was performed in triplicate with gene-specific primers and the Bio-Rad SYBR FAST iCycler qPCR kit (Kapa Biosystems) on a Biorad CFX96 real-time thermal cycler. The ΔΔC_T_ method was used to calculate expression relative to TBP.

### Simulations

To evaluate statistical properties of various approaches, we used the negative binomial (NB) pseudorandom generator to create count datasets of RNA isoform reads in 12 scenarios, each repeated three times for calculations of means and standard deviations. Our simulations were based on our Jurkat T-cell isoform data which comprise of 18290 isoforms, two conditions (resting and stimulating states) and 3 replicated libraries. Baseline data were generated by using R rnbinom function with mu = mean and size = variance of each isoform from any one of two conditions. We set two levels (A = 100 and 300) of condition (or treatment) effect on differential transcription of isoforms and linearly and randomly assigned the effect τ=UA to isoforms that are defined to be differentially transcribed where *U* is uniform variable (*U* ∈ (0, 1]), two levels of proportions of differentially transcribed isoforms: P = 10 and 30%, two levels of artificial noise proportions: Q = 10 and 30% and two levels of sample sizes: R = 3 and 5 replicate libraries. Here artificial noise (also called technical noise) indicates that the artificial noise does not come from biological system but comes from technique operations such as sequencing, mapping, assembling and pipeline analysis etc. In our simulated data, isoforms with averaged read count <5 were filtered and all replicate libraries have the same size, thus 18162 isoforms were available for analysis.

### Software package

This package for mBet t-test was written in Matlab and R languages. Matlab package consists of 9 Matlab files, two real data files and two geneid files, one simulated data files with one geneid file, and one user guidance file. This Matlab package can be found in S1 File. R mBet ttest package can be found in Bioconductor.

## Results

### t-statistic

Here we follow the notations of Baggerly et al (2003) [23]. Let *X*_*i*_ be the count of an mRNA transcript isoform of interest, herein defined by polyadenylation site identity, in library *i*. In our analysis, each of the isoforms derived from a given transcription unit is viewed as an independent entity rather than being collapsed to a single representation of the transcription unit. Let *p*_*i*_ be the true proportion of the isoform in library *i* and *N*_*i*_ be the total counts in library *i*, that is, the size of library *i*. We suppose that the proportion *p*_*i*_ of an isoform in library *i* follows a beta distribution,

$$p_{i}∼Beta\;(\alpha ,\beta )$$ (1)

while the count for an isoform will follow a binomial or negative binomial distribution. In our current study, we consider the binomial distribution instead of negative binomial distribution (see Discussion Section). Since ${\hat{p}}_{i}=X_{i}/N_{i}$ is an estimate of *p*_*i*_, the mean and variance of the estimated proportion for this isoform in library *i* are given by *α*, *β* and *N*_*i*_ (see Appendix A in S2 File).

Considering the case of a limited number of replicates, we use weight to reduce biases of the estimate of proportion p against its expectation and variance of the estimated proportion p. Supposing that we have *m* replicate libraries in a condition, the mean and variance of proportions in *m* replicate libraries can be linearly given by weights [23]:

$$E\left(\sum_{i=1}^{m}w_{i}{\hat{p}}_{i}\right)=\sum_{i=1}^{m}w_{i}E({\hat{p}}_{i})=\frac{\alpha }{\alpha +\beta }\sum_{i=1}^{m}w_{i}=\frac{\alpha }{\alpha +\beta }$$ (2A)

$$V\left(\sum_{i=1}^{m}w_{i}{\hat{p}}_{i}\right)=\sum_{i=1}^{m}w_{i}^{2}V({\hat{p}}_{i})=\sum_{i=1}^{m}\frac{w_{i}^{2}\alpha \beta }{\left(\alpha +\beta )(\alpha +\beta +1\right)}\left[\frac{1}{\alpha +\beta }+\frac{1}{N_{i}}\right]$$ (2B)

where the sum of weights over *m* replicates is constrained to be 1. Eq (2A) indicates that this combination does not change expectation of proportion p. Using a partial derivative of variance of weighted proportions with respect to weights, the solution for weight vectors can be given via use of Lagrange multipliers[23][30]:

$$w_{i}\propto {\left[\frac{1}{\alpha +\beta }+\frac{1}{N_{i}}\right]}^{-1}\;.$$ (3)

Eq (3) indicates that the weights are determined by the means and sizes of the libraries. Here two extreme cases may occur: If *α* + *β* →∞, then the weight *w*_*i*_ is proportional to the size (*N*_i_) of library *i*, meaning that the distribution of *p*_*i*_ is degenerate. In this case, there is no change in the true proportion going from sample to sample. If, on the other hand, *α* + *β* is very small, then the weights are roughly the same for all libraries. The true optimum lies somewhere in between. With the weights, the proportion *p* for an isoform read count in a condition is now estimated by

$$\hat{p}=\sum_{i=1}^{m}w_{i}{\hat{p}}_{i}$$ (4)

and its variance is also estimated in an unbiased fashion by

$${\hat{V}}^{*}=\frac{\sum_{i=1}^{m}{\left(w_{i}{\hat{p}}_{i}\right)}^{2}-(\sum_{i=1}^{m}w_{i}^{2}){\hat{p}}^{2}}{1-(\sum_{i=1}^{m}w_{i}^{2})}.$$ (5)

Since we have weights for all parameters (*α*, *β*, *p*, and V*) and *p*_*i*_ in a given condition, then an iterative search algorithm for optimal estimation of these parameters can be driven by weights (see Appendix B in S2 File).

Even if the estimation of variances of proportions in a condition is unbiased and optimized, mRNA isoforms represented by an extremely small number of observations would have very small and similar proportions in few replicate libraries. As a result, the t-values are inflated for the reason that the variances will be much smaller than the differences between means. To avoid this phenomenon, a modified estimator of variance is:

$$\hat{V}=\text{max}[{\hat{V}}^{*},{\hat{V}}^{\#}]\;.$$ (6)

In Baggerly et al [23], ${\hat{V}}^{\#}$ is given by

$${\hat{V}}^{\#}=\frac{\frac{\sum_{i=1}^{m}X_{i}}{\sum_{i=1}^{m}N_{i}}\left(1-\frac{\sum_{i=1}^{m}X_{i}}{\sum_{i=1}^{m}N_{i}}\right)}{\sum_{i=1}^{m}N_{i}}\;.$$ (7A)

From Eq (7A), we can find that $\frac{\sum_{i=1}^{m}X_{i}}{{\left(\sum_{i=1}^{m}N_{i}\right)}^{2}}$ allows the variance ${\hat{V}}^{\#}$ to become extremely small. To avoid this possibility, we modify ${\hat{V}}^{\#}$ as

$${\hat{V}}^{\#}=\frac{\frac{1+\sum_{i=1}^{m}X_{i}}{\frac{1}{m}\sum_{i=1}^{m}N_{i}}\left(1-\frac{1+\sum_{i=1}^{m}X_{i}}{\frac{1}{m}\sum_{i=1}^{m}N_{i}}\right)}{\frac{1}{m}\sum_{i=1}^{m}N_{i}}$$ (7B)

${\hat{V}}^{\#}$ in Eq (7B) is larger than that in Eq (7A). ${\hat{V}}^{\#}>{\hat{V}}^{*}$ when $\sum_{i=1}^{m}X_{i}$ is extremely small. Thus $\hat{V}={\hat{V}}^{\#}$ in case of extremely small ${\hat{V}}^{*}$.

By the above optimal estimation, we obtain ${\hat{p}}_{A}$ and ${\hat{p}}_{B}$, ${\hat{V}}_{A}$ and ${\hat{V}}_{B}$ in conditions A and B, respectively. Using these estimates, the t-statistic (similar to the Z-statistic suggested by Kar et al [19]) is found to be

$$t=\frac{{\hat{p}}_{A}-{\hat{p}}_{B}}{\sqrt{{\hat{V}}_{A}+{\hat{V}}_{B}}}$$ (8)

with degrees of freedom

$$df=\frac{{\left({\hat{V}}_{A}+{\hat{V}}_{B}\right)}^{2}}{\frac{{\hat{V}}_{A}^{2}}{N_{A}-1}+\frac{{\hat{V}}_{B}^{2}}{N_{B}-1}}$$ (9)

[23]where $N_{A}=\sum_{i=1}^{m_{A}}N_{Ai}$ and $N_{B}=\sum_{i=1}^{m_{B}}N_{Bi}$ [23]. With degree of freedom, we can obtain a p-value for each t-statistic from the t-distribution. However, since the number of experimental or technical replicates utilized in a general transcriptional profiling experiments is very small (e.g. 3 per condition), undue significance is assigned to small differences between two means. Although Eq (6) inserts another estimator of variance as a lower bound to avoid the occurrence of extremely small or zero variance, the potential for small sample sizes leading to undue significance is not excluded in Eq (8). To remove this potential, we introduce a gene-by-gene or isoform-by-isoform variable *ρ* to Eq (8) and obtain a new t-statistic

$$t_{g}{}^{*}=\frac{\rho_{g}}{\omega }\frac{{\hat{p}}_{Ag}-{\hat{p}}_{Bg}}{\sqrt{{\hat{V}}_{Ag}+{\hat{V}}_{Bg}}}$$ (10)

where $\rho_{g}=\sqrt{\zeta_{g}\psi_{g}}$, that is, *ρ*_*g*_ is defined as geometric mean of *ψ*_*g*_ and *ζ*_*g*_. *ρ* = *ψ*_*g*_ = *ζ*_*g*_ If *ψ*_*g*_ = *ζ*_*g*_. Here *ψ*_*g*_ is referred to as the “polar ratio” for measuring gap between two count sets of gene or isoform *g* (see Appendix C1 in S2 File). Equation (C1) indicates that if two count sets $X_{Ag}=\{X_{Ag1},\cdots ,X_{Agm_{A}})$ and $X_{B}=\{X_{Bg1},\cdots ,X_{Bgm_{B}})$ for an isoform do not overlap, then *ψ*_*g*_ > 1, otherwise,. *ψ*_*g*_ ≤ 1. In statistical theory, two count sets that are definitely separated have a higher probability of showing that they come from two different distributions than those that overlap. *ζ*_*g*_ is referred to as log “odds ratio” (see Appendix C2 in S2 File).

For isoform g, noise (averaged difference between maximum and minimum counts within conditions) is smaller than conditional effect or treatment effect and noise variance is small, then there would be a gap between two count sets so that *ψ*_*g*_ >1 and *ζ*_*g*_ >1, leading to *ρ*_*g*_ > 1 (we will prove this conclusion elsewhere). For example, suppose that we have count sets X_A_ = {112,122, 108,127} and X_B_ = {302, 314, 322, 328}. The noise = 22.5 and noise variance = 101 are smaller than conditional effect = 119.25. Obviously, there is a bigger gap between count sets X_A_ and X_B_. These two count sets have small noise variation. Our calculation shows *ψ* = 2.34 and *ζ =* 1.71, so *ρ* = 2.0 >1, which is consistent with observation. Another example is X_A_ = {511, 230, 754, 335} and X_B_ = {771, 842, 1014,798}. The noise = 281 is smaller than conditional effect = 398.8 but noise variance = 32214.3 is larger than conditional effect. One can visually see that these two count sets have a gap but their homogeneity is poor (big noise variance). Our calculation that shows *ψ* = 1.02>1, *ζ* = 0.38<1 and *ρ* = 0.623<1 is again agreeable with observation. The t*-statistic is potentially preferable to *t*-statistic in two aspects: (1) isoforms with small counts are not easily found to have differential expression and (2) t-values with *ρ*_*g*_ > *ω* are inflated but those with *ρ*_*g*_ < *ω* are shrunken. *ω* is a threshold. *ω* directly affects the performance of mBeta t-test: the larger *ω* is, the more t*-values with *ρ*_*g*_ ≤ *ω* are shrunken, the less those with *ρ*_*g*_ > *ω* are inflated. *ω* is determined by the null simulation based on the real data. Here we use the simulated null data to perform our mBeta t-test with setting *ρ* = 1 and *ω* = 1, find false DE isoforms, calculate their *ρ* values, then order them from the smallest to the largest, *ρ*_1_ < *ρ*_2_ < ⋯ *ρ*_*j*_ < ⋯ < *ρ*_*k*_, and calculate quantiles. We set *q*_1_ = 1/*k*, *q*_2_ = 2/*k*, ⋯, *q*_*j*_ = *j*/*k*, ⋯ *q*_*k*_ = 1 where *k* is number of false discoveries in a null simulated dataset. Setting *q*_*j*_ ≥ 0.85, then we choose *ω* = *ρ*_*j*_ value. This means that 85% false discoveries would have *ρ*_*j*_ ≤ *ω* and be excluded. This process is done on all given simulated null datasets. We choose $\bar{\omega }=\frac{1}{S}\sum_{s=1}^{S}\omega_{s}$ over S simulated null datasets. The p-value for each t*-value can be obtained from t-distribution using degrees of freedom given or by performing the bootstrap [31] (see Appendix D in S2 File).

### Comparison of mBeta t-test to existing statistical methods

We used the 12 scenario stimulation datasets (see Simulation in MATERIALS AND METHODS) to compare our method to the five top statistical methods for identifying isoforms differentially transcribed between two given conditions. The five statistical methods chosen here are Beta t-test [23], empirical Bayesian (baySeq)[25], edgeR Exact test [18,32,33], edgeR GLM [4], and DESeq [17]. The baySeq method was implemented in R package baySeq and the edgeR Exact test and edgeR GLM methods in R package edgeR[34]. DESeq was implemented by R package DESeq[17]. The Beta and mBeta t-test methods were performed in Matlab. We chose FDR cutoff = 0.05 as acceptable level for differential expressions (DE) of isoforms because the FDR cutoff of 0.05 is widely used in multiple tests, especially, in genome-wide studies. We counted isoforms identified to be differentially transcribed by these methods and false discoveries and calculated means and standard deviations (SD) of the numbers of findings and true FDRs for each method chosen and then summarized them in Tables A-C in S3 File. For small condition effect (A = 100) or low artificial noise proportion (Q = 10%) or low proportion (P = 10%) of DE isoforms, the Beta t-test method had higher power. In the case of low P or small A, mBeta t-test, baySeq, edgeR Exact test and edgeR GLM had similar powers, while in higher P and larger A scenarios, Beta and mBeta *t*-test had lower powers than baySeq, edgeR Exact test and edgeR GLM. In all 12 scenarios (Tables A-C in S3 File), DESeq had very low powers. This is because DESeq always had extreme overestimation of FDRs, indicating that DESeq is a very conservative method that would miss many true differentially expressed isoforms in practice. Beta t-test had much higher true FDRs than its estimated FDRs in all these scenarios, meaning that in the findings of the Beta t-test method, there would be many more false discoveries than estimated, so this is not a conservative method. baySeq showed higher powers in low artificial noise proportion (Tables A and C in S3 File) but it also had higher true FDRs than estimated FDRs in most cases. In high artificial noise proportion (Q = 30%) scenario, baySeq performed well (Table C in S3 File). edgeR GLM showed high powers in all 12 given scenarios but its true FDRs were much larger than estimated in 9 scenarios (Tables A-C in S3 File), suggesting that this method is also not conservative. In low DE isoform proportion (P = 10%), low artificial noise proportion (Q = 10%) or small condition effect (A = 100) scenario, edgeR Exact test performed poorly because its true FDRs were much larger than its estimated values in most cases, however, in high P(30%), high Q(30%) and large A (300) scenarios, edgeR Exact test had good performance. Similarly to DESeq, mBeta t-test also had lower true FDRs than its estimated values in all 12 given scenarios but it had much higher power than DESeq (Tables A-C in S3 File), showing that the mBeta t-test method is conservative and powerful.

Stability is an important property of a statistical method. To rate stabilities of these statistical methods in performance, we used standard deviations (SD) of finding numbers and true FDRs listed in Tables A-C in S3 File as criterion. Small SD means that this method has a small fluctuation and hence a high stability in identification of DE isoforms, while larger SD indicates that it has a bigger fluctuation and hence lower stability. Thus, for each scenario we set, we ordered these methods by using SD from the smallest to the largest, assigned order scores (from 1 to 6) to them and averaged their order scores over 12 scenarios. Thus, the order score can be used to measure relative stability of a method: the smaller order score, the higher stability. Table 1 summarizes the results of stability analysis. For findings, mBeta *t*-test had the highest stability, while edgeR GLM had the lowest stability. Beta *t*-test, baySeq, DESeq and edgeR Exact test got similar order scores and so they had proximate stabilities. For true FDR, as expected, DESeq showed the highest stability. mBeta *t*-test was in the second highest rank. edgeR GLM and Beta t-test were lowest. edgeR Exact test and baySeq showed similar stabilities.

**Table 1:** Stability analysis of statistical method performance on simulated data in 12 scenarios.

Standard Deviation of Findings		Standard Deviation of True FDRs	
Method	Averaged Order Score	Method	Averaged Order Score
mBeta t-test	2.33	DESeq	1.83
edgeR Exact test	3.17	mBeta t-test	2.50
Beta t-test	3.25	baySeq	3.00
baySeq	3.42	edgeR Exact test	3.50
DESeq	3.83	Beta t-test	5.08
edge R GLM	5.00	edgeR GLM	5.08

**Fig 1:**
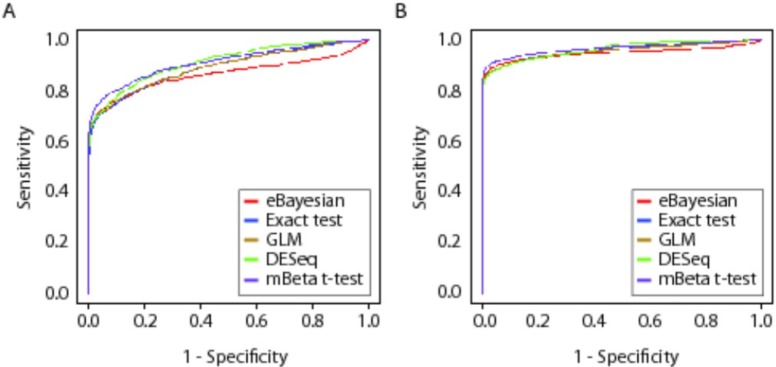
ROC Comparison among Statistical Methods for Differential Expression Analysis of NGS Data. ROC curves of the baySeq, edgeR Exact test, edgeR GLM, DESeq and mBeta t-test methods were constructed from simulated datasets. Sensitivity is defined as the true positive fraction (TPF) and specificity is defined as the false positive fraction (FPF). **A:** Simulated data from scenario 1 (proportion of differentially expressed isoforms = 10%, technical noise proportion = 10%, treatment effect A = 100, and sample size = 3). **B:** Simulated data from scenario 4 (proportion of differentially expressed isoforms = 30%, technical noise proportion = 10%, treatment effect A = 300, and sample size = 3).

To globally evaluate each of the six statistical methods, we employed the simulated data in scenarios 1 and 4 to generate Receiver Operating Characteristic (ROC) curves. Fig 1 shows the ROC curves did not reveal a substantial difference among the methods. The mBeta t-test method performed best at 1-specificity value < 0.3 in scenario 1 (Fig 1A) or < 0.5 in scenario 4 (Fig 1B). DESeq produced a slightly higher sensitivity than mBeta t-test when 1-specificity > 0.3 in scenario 1 (Fig 1A) or > 0.5 in scenario 4 (Fig 1B). Comparatively, edgeR Exact test and edgeR GLM had almost the same curve. The baySeq method performed poorly relatively (Fig 1).

Next is to comprehensively rate these statistical methods. We define efficiency (*w*) of a statistical method as

w=ϕφ (11)

where $\phi =\frac{N_{f}}{N_{P}}$. Here *N*_*f*_ represents the number of isoforms found to be truly and differentially expressed by a statistical method, *N*_*P*_ = *NP* is a given number of differentially expressed isoforms in *N* isoforms given in the simulated data and P, the proportion of DE ioforms given in the simulation. *φ* is index. Given FDR cutoff α, *φ* = 1 if true FDR < α or *φ* = 0 otherwise. Thus, *ϕ* is used to measure power (ability or probability) of a statistical method for identifying a differentially expressed isoform while *φ* measures conservativeness of this method under a given FDR cutoff. Efficiency is power with given FDR cutoff α and similar to that with given significance level α in single hypothesis test. The performance of a method must be evaluated by its power and conservativeness. If a method has high power to find DE isoforms with low degree of conservativeness, its findings would then be unreliable and incredible; if a method has low power with high degree of conservativeness, the method would then loss many true positives. So such two types of statistical methods would have low efficiencies in identification of DE isoforms.

Table 2 lists efficiencies of the six statistical methods in several scenarios. From Table 2, one can find that the baySeq and edgeR GLM methods had higher efficiencies in 3 replicate libraries than in 5 replicate libraries. This is because in the case of five replicate libraries, the two methods underestimated their FDR at cutoff *α* = 0.05 (Tables A-C in S3 File) so that they lost conservativeness. Beta t-test had efficiency of zero in all scenarios. edgeR Exact test had lower efficiencies in scenarios with low P, low Q, small A, and 3 replicates than in scenarios with high P, high Q, large A, and 5 replicates. For both the DESeq and mBeta *t*-test approaches, the efficiencies were greatly raised with increment of sample size. This is due to the fact that the two methods increased their powers without losing conservativeness when sample size increased. Similar results also can be observed in condition effects A = 100 versus A = 300. The efficiencies of the two methods did not significantly respond to change in proportion of DE isoforms and artificial noise proportion. However, in all simulated scenarios, the mBeta t-test method had the highest efficiencies.

**Table 2:** Efficiencies of statistical method performance on simulated data in 12 scenarios.

Scenario factors	baySeq		edgeR Exact test		edge GLM		DESeq		beta t-test		mBeta t-test	
	mean	SD	mean	SD	mean	SD	mean	SD	mean	SD	mean	SD
3 replicate libraries	0.487	0.321	0.373	0.407	0.276	0.388	0.576	0.151	0.000	0.000	0.692	0.120
5 replicate libraries	0.000	0.000	0.463	0.538	0.000	0.000	0.813	0.151	0.000	0.000	0.871	0.072
proportion of DE genes/isoforms = 10%	0.310	0.353	0.132	0.324	0.000	0.000	0.638	0.094	0.000	0.000	0.753	0.144
proportion of DE genes/isoforms = 30%	0.328	0.368	0.498	0.430	0.169	0.318	0.668	0.189	0.000	0.000	0.763	0.150
technical noise proportion = 10%	0.318	0.368	0.212	0.423	0.213	0.426	0.629	0.198	0.000	0.000	0.730	0.119
technical noise proportion = 30%	0.328	0.368	0.498	0.430	0.169	0.318	0.668	0.138	0.000	0.000	0.763	0.150
condition effect = 100	0.383	0.303	0.235	0.376	0.095	0.232	0.543	0.198	0.000	0.000	0.661	0.121
condition effect = 300	0.267	0.413	0.570	0.448	0.273	0.423	0.767	0.164	0.000	0.000	0.843	0.077

Since simulated data are generally made from a known distribution and all factors impacting differential expression are well controlled, simulation evaluation has a limited significance for application of the methods to the real world data. However, since everything, in particular, noise distribution in real world data is unknown, it is impossible to conduct a direct evaluation of statistical methods by comparison of true FDRs to estimated ones. We thus employed an indirect way for this comparison using two real transcriptomic datasets of our laboratory.

We utilized a PAS-seq [10] approach to assess relative expression changes occurring upon antigen receptor stimulation of the Jurkat CD4^+^ T cell line, comparing three experimental replicates of non-stimulated cells to an equivalent number of replicates cells stimulated for 48 hours. Following mapping, normalizing, and filtering of the data sets (see MATERIALS AND METHODS), we observed 13409 mRNA isoforms (again, with the working definition of cleavage and polyadenylation site usage) in 9572 genes. Relative expression of an mRNA isoform was measured by using read counts of this mRNA isoform and gene expression was represented by sum of read counts over all mRNA isoforms mapping to a known transcription unit.

We used these two transcriptomic datasets to evaluate these six chosen statistical methodologies. The baySeq method returned an NA result in the gene data and no results in the isoform data after running for over two days on a 12-core Mac server. In contrast, the edgeR GLM method identified 4376 (45%) genes and 5039 (37%) isoforms, respectively, of being differentially expressed at FDR cutoff of 0.05. This high rate of findings in both gene and isoform datasets is doubtable. Highlighting its high degree of conservativeness and stringency, DESeq found only 261 (3%) differentially expressed genes and 287 (2%) differentially expressed isoforms, respectively. The numbers of differentially expressed genes and isoforms identified by the edgeR Exact test, Beta *t*-test, and mBeta *t*-test methodologies fell in between these two extremes (Table 3) and were examined further.

**Table 3:** Performances of Three Statistical Methods on Jurkat PAS-seq Data.

Data Type		edge R Exact test	mBeta t-test^a^	Beta t-test
Gene Count Data	# of DE Genes Found	799	1774	2515
	Estimated FDR	0.0499	0.0499	0.0489
	Least True FDR	0.2753	0.0056	0.3062
Isoform Count Data	# of DE Isoforms Found	1029	1981	3025
	Estimated FDR	0.0499	0.0498	0.0499
	Least true FDR	0.2799	0.0101	0.3530

a: *ω* = 1

**Fig 2:**
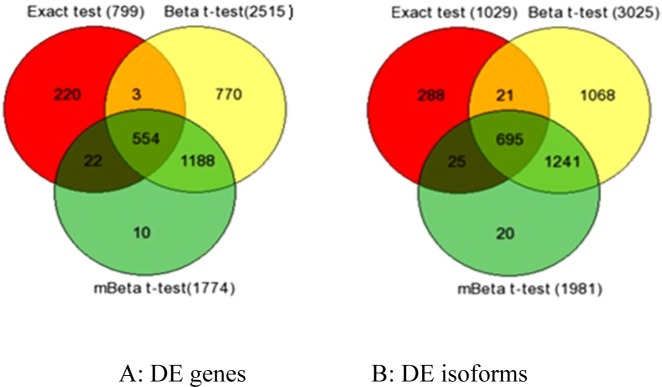
Differential Expression Venn diagram. Numbers of edgR Exact test findings, Beta t-test findings and mBeta t-test findings are listed in red, yellow and green cycles, respectively. In DE genes (A) and DE isoforms (B), 98% of mBeta t-test findings are the same with Beta t-test findings, the edgeR Exact test method has about 70% of findings that overlap with Beta t-test and mBeta t-test findings.

In the next step, we compared the findings of the edgeR Exact test (799 DE genes), Beta *t*-test (2515 DE genes), and mBeta *t*-test (1774 DE genes) using Venn diagram. The three methods commonly identified 554 genes having differential expressions (Fig 2A). Outside of this common set, 22 differentially expressed genes were commonly identified by the edgeR Exact test and mBeta t-test, whereas three differentially expressed genes were commonly identified by the edgeR Exact test and Beta t-test. Analysis of the isoform dataset revealed a similar result (Fig 2B).

**Fig 3:**
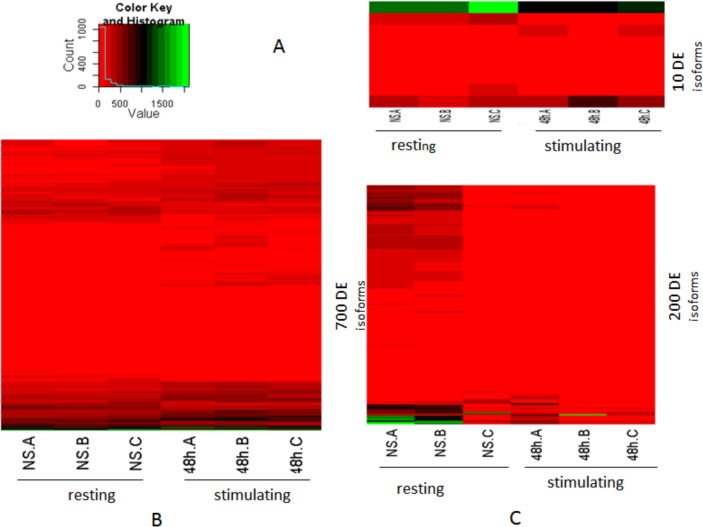
Heatmaps of Expressions of Transcripts Identified Only by Single Statistical Methods from Real Transcriptomic Datasets. **A:** 10 Isoforms were specifically called differential expression by mBeta t-test. **B:** 700 isoforms were specifically found to be differentially expressed by Beta t-test. **C:** 220 DE isoforms were defined only by edgeR Exact test. NS: non-stimulated cells. 48h: stimulated cells via antigen receptor for 48 hours. A, B, and C are replicates.

Within this comparison, if a gene or isoform is found to be differentially expressed by only a single method, then it is highly possible that this differentially expressed gene or isoform is a false discovery. Fig 3 visualizes heat maps of the ten genes specifically identified by the mBeta *t*-test method (Fig 3A), the 770 genes identified solely by the Beta *t*-test (Fig 3B), and the 220 genes identified only by the edgeR Exact test (Fig 3C). Indeed, the genes identified only by a single method do not display obvious differences in expression between the non-stimulated and stimulated states. We defined no share ratio of findings (*m*_*i*_ / *M*_*i*_ where *m*_*i*_ is number of method *i*-specific findings, and *M*_*i*_ is numbers of findings identified by method *i*) as the least true false discovery rate (where the least true FDR corresponds to the q-value defined by Storey et [35]). Using this indirect method, we obtained the least true FDRs for the findings of the edgeR Exact test, mBeta *t*-test and Beta *t*-test methods, respectively, in our real PAS-seq datasets (Table 3). From Table 3, one can see that edgeR Exact test and Beta t-test severely underestimated their FDRs, while the mBeta *t*-test still overestimated FDR in its findings. This is consistent with the results obtained from the simulated data.

**Fig 4:**
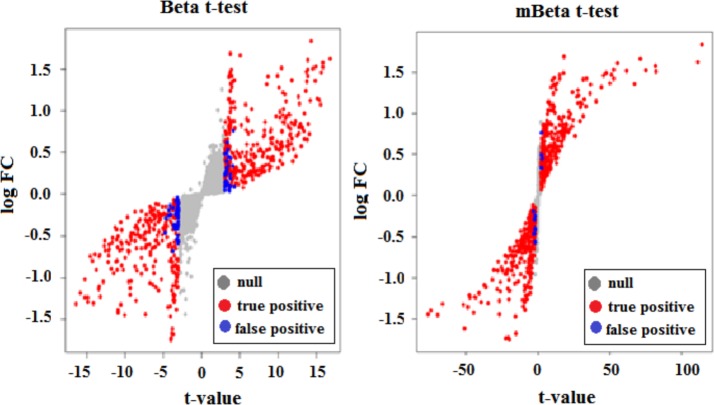
Plot of Log Fold Change versus *t*-statistics. *t*-values were obtained by the two Beta *t*-test methods from the simulated data in which values τ = 100*U* were assigned to 10% of genes for differential expression between two given conditions each with three replicates. LogFC on y-axis represents log fold change in expression. In the Beta t-test method (A), *t*-value was distributed in the interval between -16 and 16 and acceptable region for non-differential expression was spanned from -4.0 to 4.0. Many false positives (blue dots) were found in two rejection areas. But in the mBeta t-test method (B), the interval for t-values was enlarged to span from -70 to 120 and the acceptable region for non-differential expression was strongly compressed into a narrow interval close to zero so that few false positives (blue dots) were found in rejection areas.

To see why mBeta t-test method had so high efficiency and stability, we used plot of log fold change against *t*-value to compare Beta *t*-test to mBeta *t*-test (see Fig 4). In Beta *t*-test, *t*-value distributes from -16 to 16, while in mBeta *t*-test, *t*-value distributes from -70 to 120. On the other hand, Fig 4 shows that Beta *t*-test has acceptable region of -4 to 4 and many false discoveries scattered in two rejection regions nearby acceptable region while mBeta *t*-test significantly compresses acceptable region to a very narrow interval close to zero such that the false discoveries (blue dots) are significantly reduced.

### Experimental validation

**Fig 5:**
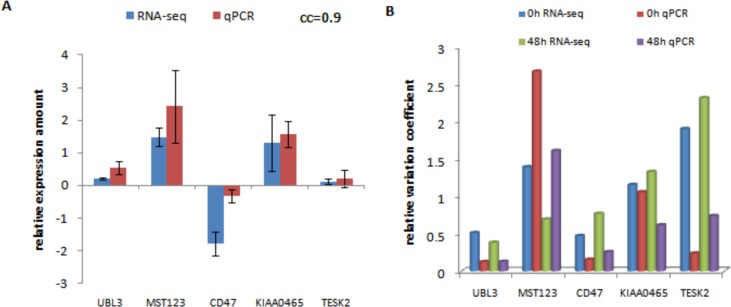
RT-PCR Validation of Differential Expression. **A:** comparison of relative expressions of five genes in PAS-seq to their relative expressions in qRT-PCR. In PAS-Seq data, relative expression of a gene is defined as $d_{g}/\bar{d}$ where $d_{g}={\bar{n}}_{gt}-{\bar{n}}_{g0}$, $\bar{d}$ is the averaged value of *d*_*g*_ over three replicates, g = *UBL3*, *MST123*, *CD47*, *KIAA0465* or *TESK2*, $\bar{n}$ is the averaged count of reads over three replicates and t = 48 hours of stimulation. The-ΔΔC_T_ method was used for representation of qRT-PCR data and *TBP* (TATA binding protein) was used as a reference. **B:** Relative expression variation coefficients of the five genes in PAS-seq and qPCR data. Relative expression variation coefficient is defined as $VC_{gt}/{\bar{VC}}_{t}$ where $VC_{gt}={\bar{n}}_{gt}/s_{gt}$ and ${\bar{VC}}_{t}$ is the averaged variation coefficient over all selected genes at time t of stimulation and *s*_*gt*_ is sample standard deviation of gene g at stimulation time t.

To validate the findings of the mBeta t-test, we carried out quantitative PCR experiments using RNA derived from resting Jurkat T-cells and Jurkats stimulated via the antigen receptor for 48 hours. We randomly chose genes that were identified by the mBeta t-test method to be increase, decrease or not change in expression. The RNA-seq and qPCR data were compared using both relative differences between stimulation and rest and relative variation coefficient (VC). Genes UBL3, MST123 and KIAA0465 that were up-regulated to respond to stimulation (blue columns in Fig 5A) in RNA-seq data also displayed positive response to stimulation in qPCR data (red columns in Fig 5A). Gene CD47 negatively responded to stimulation in both datasets while gene TESK2 was not detected to have significantly difference between stimulation and rest in these two datasets. In expression direction and relative expression amount, these two datasets show cc = 0.9 (Pearson correlation coefficient) (Fig 5A), suggesting that our transcriptomic data are agreeable with qPCR data. Relative VC analysis revealed that the *UBL3* gene had small noise variation in these two datasets while the expresssion variation in the *TESK2* gene across 3 replicates was relatively high (Fig 5B). This explains why the *UBL3* gene was identified to be differentially expressed but the TESK2 gene was not, even though both of these genes had comparatively small counts of reads in the transcriptomic data.

## Discussion

Although our simulation data were made from the NB distributions, Beta and mBeta t-test worked well **if we do not consider whether estimated FDRs were or not over true FDR”**. The baySeq, edgeR GLM, edgeR Exact test, and DESeq approaches are merely based on the NB distribution. Therefore, for real datasets whose distributions are often unknown, mBeta t-test will perform well. For example, as seen in RESULTS Section, baySeq and edgeR GLM, DESeq performed poorly on our isoform and gene data, while edgeR Exact test, Beta t-test and mBeta t-test worked even though their results had big differences. We also applied these methods to our another real transcriptomic data containing 10299 genes (not yet published), the results show that except baySeq had very low power (it just found 165 DE genes), edgeR GLM, edgeR Exact test and mBeta t-test identified respectively 780, 733 and 711 DE genes and hence performed very similarly. These suggest that baySeq and edgeR GLM may be specific to the NB distribution.

In addition, in genome-wide data, especially, in transcriptomic data, sample sizes usually are very small, for example, 4 or 3 replicate libraries in each condition due to high cost, limitation of biological resources and experimental conditions. Small sample size would easily lead to a fudge effect [27,36]. In count data containing more than ten thousands of isoforms, for example, 3-replicate count sets would have larger probability of showing small noise variation than 5-replicate count sets. On the other hand, in transcriptome-wide data, small count data have more chance to be weakly fluctuated by noise and to form extremely small within-group variances than big count data, giving rise to inflating *t*-statistics. For general statistical methods, the genes or isoforms with small count data derived from small samples would easily be found to be differentially expressed between conditions.

To address this problem, many methods developed for identifying differentially expressed genes in microarray data introduce a constant to shrink statistics. For example, in SAM [28], the two-sample *t*-test is modified as a d-statistics by adding a minimized coefficient of variation s_0_ to denominator. In the regularized *t-*test method [37], the two-sample *t*-test is modified by combining gene-specific variance with global average variance. However, since these approaches shrink the t-statistics for all genes, the power of these approaches is markedly decreased. Tan et al [27] developed a conditional shrinking method to address the problem of inflated *t*-statistics, but this approach cannot be introduced into Beta *t*-test since Beta *t*-test is based on differences in frequencies (proportions) of tags between conditions [23].

Baggerly et al [23] employed a weighting and iteration strategy to look for an optimal estimation of parameters and frequency is assumed to follow beta distribution for a tag in a condition and furthermore developed a new *t*-test, we called Beta t-test. Weight and optimization are a useful strategy for excluding artificial or technical noise in count data. Although Baggerly et al [23] recognized small counts leading to inflation of *t*-tests and tried to avoid the problem of *t*-value inflation using alternative variance given in Eq (7A), our practice demonstrated that Eq (7A) does not substantially release the fudge effect. For this reason, we modified the alternative variance by utilizing means of total counts over all replicate libraries in a condition for those isoforms with very small counts. Analytically, it can be seen that the alternative variance defined in Eq (7B) is larger than that in Eq (7A). Our simulation really showed that the above small-count effect on testing for differential expressions of isoforms was mostly reduced by our modified alternative variances.

In order to eliminate effect of small sample size, we introduced a gene-or isoform-specific variable *ρ* into the Baggerly et al.s’ [23]Beta *t*-test. *ρ* is used to measure overlap between two count sets. If two count sets more overlap and/or have bigger within-group variances, then *ρ* becomes smaller; if two count sets separate and have small noise variation, then *ρ* >1. The larger gap between two count sets is, the larger *ρ* is. In theory, two count sets that are separated have higher probability of showing that they come from two different distributions than those that overlap. Besides, if noise variation within group is large, then *ζ* is small, which makes *ρ* become small. Thus, *ρ* shrinks *t*-values of overlapped count sets and inflates *t*-values of separated count sets with small noise variation. As seen in Tables A-C in S3 File, compared to the Beta *t*-test method, our mBeta *t*-test approach did not obviously decrease its power but significantly reduce false discovery rate so that it has higher efficiencies. Considering sample size effect, we set a threshold *ω* for *ρ*. That is, *t*-value is inflated with *ρ* > *ω* or shrunken with *ρ* < *ω*. As a result, almost all of *t*-values with *ρ* < *ω* are compressed into a very short interval close to zero but those with *ρ* > *ω* are enlarged and a mixed *t*-value region containing truly positives and false positives becomes very narrow (Fig 4). Since p-value only depends on *t*-value given degree of freedom, p-values with inflating *t*-values are reduced while those with shrinking *t*-values become larger, so very few false positives were found by mBeta *t*-test (Fig 4). Threshold *ω* depends on sample size. The larger sample size is and the smaller *ω* is. However, when sample size is large, *ω* becomes very small, ability of *ρ* controlling false discoveries becomes very weak because the gaps between two datasets are vanished and there is not fudge effect in such data.

ROC analysis is popularly used to evaluate statistical methods [25] [33]. However, the ROC curves in Fig 1 did not reveal substantial differences among the methods we assessed, particularly in Scenario 4. We thus extended our evaluation of these methods to compare the true and estimated FDRs in each case. Analysis of the simulation data revealed that the baySeq and edgeR GLM methods performed well in ideal NB distributions with lower proportions of differentially expressed isoforms, smaller condition effects, and smaller numbers of replicate libraries. However, performances of these methods are not very well when the sample sizes, the proportion of differentially expressed isoforms, or the condition effect was increased. We thus use efficiency to evaluate performances of statistical methods, which is comprised of power (the ability of finding differentially expressed mRNA transcripts or isoforms) and conservativeness of FDR estimation (the reliability of findings). Both are equally important—a statistical method with high power but no conservativeness yields unreliable findings while a very conservative method with low power would miss many truly differentially expressed isoforms. The two types of methods would have low efficiency. In our compared methods, the Beta t-test and DESeq methods are two extreme methods, Beta t-test has very high power but its false discoveries are highly over its estimated ones, so its findings are not creditable while DESeq has very low power with so high degree of conservativeness. Recently, authors have recognized that DESeq has this problem and proposed a new DESeq, called DESeq2 [38]. We compared DESeq2 to mBeta *t*-test using three repeated simulation NB datasets in two scenarios: (1) 10070 genes, 3 replicate libraries in each of two conditions, 20% of genes with technical noise and 10% of DE genes and difference effect value = 100U where U is uniform variable and (2) 11341 genes, 3 replicate libraries in each condition, 20% of genes with technical noise, 30% DE genes, and difference effect value = 300U. The results show that in scenario(1), under estimated FDR cutoff = 0.05, DESeq2 found, on average, 578 DE genes with SD = 110.2 and true FDR = 0.019 with SD = 0.0133, mBeta *t*-test found, on average, 651 DE genes with SD = 67.7 and true FDR = 0.031 with SD = 0.0142; in scenario(2), DESeq2 identified, on average, 2511 DE genes with SD = 329.7 and had true FDR = 0.0202 with SD = 0.0051 while mBeta *t*-test obtained, on average, 2679 DE genes with SD = 191.63 and true FDR = 0.00739 with SD = 0.0015. Compared to the results in Tables A-C in S3 File, DESeq2 significantly increases power with still keeping high degree of conservativeness. In addition, DESeq2 was applied to our real gene transcriptomic data and found 1243 DE genes, of which 1023 DE genes (83%) are the same with our mBeta *t*-test findings. This furthermore demonstrates performance of our mBeta *t*-test method.

Since differential splicing data are count data of RNA isoform reads and also obtained from a few replicate libraries, our method can efficiently be applied to the genome-wide detection of differential splicing events in genes responsive to drug stimulation or to change in cell status.

Finally, while the mBeta *t*-test method described above is more computationally intensive than many of the previously described approaches, it is significantly less intensive than the baySeq method, which yielded no results in our data. The computational intensity of the mBeta *t*-test method is a function of the algorithm running literately to look for an optimal estimation of weight and beta and alpha parameters for each gene or isoform. However, for the described datasets (~9,500–18,000 features), the algorithm finishes its work in 15 minutes on a standalone server if bootstrap is not utilized to calculate p-values.

## Conclusions

In count data of RNA reads from small samples, the mBeta *t*-test method not only substantially reduces false discoveries in differential expression identification so that it had high work efficiencies but also has high stability in finding isoforms of being differentially expressed and in true FDR. Simulated and real data strongly suggest that the mBeta *t*-test method would offer us a creditable and reliable result of statistical analysis in practice.

## Supporting Information

S1 File This package contains 6 m files for execution of mbeta t-test. Among them mbttest.m is main program. In addition, the package also contains 6 text files, one csv file and excel file. The 6 text files are two real data files and two the geneid files, one simulated file, one geneid file. These files provide examples for how to perform mbeta t-test.
(RAR)

S2 File Appendix A gives expectation and variance of frequency estimate in beta distribution. Appendix B gives optimal estimations of α, β, frequency, weight and variance using iteration algorithm. Appendix C gives formulae of *ψ* and *ζ* in rho calculation. Appendix D gives p-value calculation using bootstrap method.
(DOC)

S3 File Results of performing six statistical methods on simulated data of 11341 genes and two conditions in simulation Scenarios 1–12. (DOC)
