# Supplementary material for: A Powerful Statistical Approach for Large-Scale Differential Transcription Analysis
Source: PLoS One. 2015 Apr 20;10(4):e0123658. doi: 10.1371/journal.pone.0123658 (PMC4404056; doi:10.1371/journal.pone.0123658)
Supplement: S2 File — Appendix B gives optimal estimations of α, β, frequency, weight and variance using iteration algorithm. Appendix C gives formulae of ψ and ζ in rho calculation. Appendix D gives p-value calculation using bootstrap method. (DOC) [file pone.0123658.s002.doc]

**Appendix A**

$E(\hat{p}_{i})=\frac{\alpha}{\alpha+\beta}$ (A1)

(A2) **Appendix B**

To estimate parameters of beta distribution from count data, we employ an iterative algorithm to realize optimal estimation given by Baggerly et al (2003). The iteration steps are as follows

At the initial step, we set

$w_{i}^{(0)}=\frac{N_{i}}{\sum_{i=1}^{m} N_{i}}$ ,$\hat{p}_{i}=\frac{X_{i}}{N_{i}}$, and . (B1)

At the jth step, we have

, (B2)

, (B3)

$w_{i}^{(j)}\propto\frac{\left(\alpha^{(j)}+\beta^{(j)}\right)N_{i}}{\alpha^{(j)}+\beta^{(j)}+N_{i}}$. (B4)

, (B5)

. (B6)

The jth step is looped by iteration.

At the final step, the iteration stops and exits out of loop when

$d^{(j)}=w_{i}^{(j)}-w_{i}^{(j-1)}<\Delta$ (B7)

where $\Delta$is a given tolerant threshold.

**Appendix C**

We use $\psi$to measure overlap intensity between two datasets for a gene or isoform and use $\zeta$ to score degree of homogeneity among replicate observations:

$\psi=\max\left[\frac{\min(X_{A})}{\max(X_{B})+1},\frac{\min(X_{B})}{\max(X_{A})+1}\right]$ (C1)

. (C2)

where $\max(X)+1$ avoids no definition when max(X) = 0 and where $\delta_{i=j}=1$and $\delta_{i\neq j}=0$. To allow $\xi$to have the same order with$\psi$, we make log transformation of$\xi$: $\zeta=\log(1+\xi)$> 0. $0<\psi<\infty$and $0<\zeta<\infty$. For a gene or isoform, $\psi$<1 means two datasets overlap, $\psi$>1 indicates a gap between two datasets. $\zeta$<1 means that replicate observations have poor homogeneity while $\zeta$> 1 implicates better homogeneity among the replicate observations. The examples can be seen in text.

Appendix D

The p-value for each t^*^-value can be obtained by performing the following bootstrap (Storey et al. 2005):

Step 1: Resampling in real count data to create B bootstrap datasets.

Step 2: Employ the iteration algorithm in Appendix B to optimally estimate proportions and variances for each isoform in groups X_A_ and X_B_.

Step 3: Calculate $t_{bj}^{*}$-statistic from bootstrap data and obtain B sets of M $t_{b}^{*}$-statistics.

Step 4: p-value for the real t^*^-statistic of isoform k is calculated by

$p_{k}=\frac{\sum_{b=1}^{B} n_{bk}(t_{bj}^{*}>t_{k}^{*}>0,j=1,...,M)}{BM}$or $p_{k}=\frac{\sum_{b=1}^{B} n_{bk}(t_{bj}^{*}<t_{k}^{*}<0,j=1,...,M)}{BM}$

where $n_{bk}$is number of $t_{bj}^{*}>t_{k}^{*}>0$or $t_{bj}^{*}<t_{k}^{*}<0$over M isoforms.
