# Supplementary material for: A Powerful Statistical Approach for Large-Scale Differential Transcription Analysis
Source: PLoS One. 2015 Apr 20;10(4):e0123658. doi: 10.1371/journal.pone.0123658 (PMC4404056; doi:10.1371/journal.pone.0123658)
Supplement: S3 File — (DOC) [file pone.0123658.s003.doc]

**Table A:** Results of performing statistical methods on simulated data of 11341 genes and two conditions in simulation Scenarios 1 – 4, varying proportion and condition effects in the context of low (10%) artificial noise and three replicates for each condition.

| scenario | Statistical  Method | numbers of findings | | estimated FDR |  | true FDR | |
| --- | --- | --- | --- | --- | --- | --- | --- |
|  |  | mean | stdev |  |  | mean | stdev |
| Scenario 1 | eBayesian | 684.3 | 20.74 | 0.049901661 |  | 0.023415632 | 0.005316 |
|  | Exact test | 740.3 | 34.70 | 0.049290389 |  | 0.086608166 | 0.009598 |
|  | GLM | 753.3 | 36.55 | 0.049609439 |  | 0.096184239 | 0.009912 |
|  | DESeq | 552.0 | 24.24 | 0.049794642 |  | 0.007797678 | 0.002519 |
|  | beta t-test | 800. 7 | 4.93 | 0.049847667 |  | 0.144082436 | 0.010284 |
|  | mBeta t-test | 726.0 | 3.46 | 0.048788614 |  | 0.037659939 | 0.008904 |
| Scenario 2 | eBaysian | 990. 7 | 17.61 | 0.04970673 |  | 0.06424989 | 0.004605 |
|  | Exact test | 1003.0 | 28.68 | 0.04971551 |  | 0.089023333 | 0.002429 |
|  | GLM | 1015.3 | 31.34 | 0.049347345 |  | 0.097436683 | 0.0047 |
|  | DESeq | 855.0 | 14.42 | 0.048391895 |  | 0.011703212 | 0.005338 |
|  | beta t-test | 1000.0 | 25.23 | 0.049856733 |  | 0.155683147 | 0.008473 |
|  | mBeta t-test | 957.3 | 12.50 | 0.048912878 |  | 0.035496106 | 0.007327 |
| Scenario 3 | eBayesian | 2271.3 | 49.52 | 0.049921947 |  | 0.027993743 | 0.002909 |
|  | Exact test | 2299.3 | 36.69 | 0.049936596 |  | 0.053499728 | 0.002031 |
|  | GLM | 2316. 7 | 35.21 | 0.049880522 |  | 0.058151276 | 0.003686 |
|  | DESeq | 1815. 7 | 32.02 | 0.049790935 |  | 0.006585122 | 0.00228 |
|  | beta t-test | 2323.0 | 46.11 | 0.049676633 |  | 0.071477617 | 0.009545 |
|  | mBeta t-test | 2093.0 | 32.90 | 0.049291886 |  | 0.012110596 | 0.002648 |
| Scenario 4 | eBayesian | 3024.0 | 166.91 | 0.04991893 |  | 0.070821217 | 0.01752 |
|  | Exact test | 2880.3 | 145.08 | 0.049928763 |  | 0.044367555 | 0.008163 |
|  | GLM | 2896.0 | 150.06 | 0.049981628 |  | 0.048324792 | 0.009311 |
|  | DESeq | 2517. 7 | 177.80 | 0.049904472 |  | 0.005894893 | 0.001385 |
|  | beta t-test | 2913.0 | 40.92 | 0.049404633 |  | 0.073357708 | 0.007807 |
|  | mBeta t-test | 2789.0 | 41.76 | 0.049425529 |  | 0.008624077 | 0.002894 |

Scenario 1: P=10%, Q=10%, A=100, R=3

Scenario 2: P=10%, Q=10%, A=300, R=3

Scenario 3: P=30%, Q=10%, A=100, R=3

Scenario 4: P=30%, Q=10%, A=300, R=3

P = proportion of differentially expressed isoforms.

Q = artificial noise proportion.

A = condition effect.

R = number of replicates.

$\omega=1$for mBeta t-test method in Scenarios 1-4. Underestimated FDR is indicated in red.

**Table B**: Results of performing statistical methods on simulated data of 11341 genes and two conditions in simulation Scenarios 5 – 8, varying proportion and condition effects in the context of higher (30%) artificial noise and three replicates for each condition.

| scenario | Statistical  methods | number of findings | | estimated FDR |  | true FDR | |
| --- | --- | --- | --- | --- | --- | --- | --- |
|  |  | mean | stdev |  |  | mean | stdev |
| Scenario 5 | eBayesian | 536.0 | 19.31 | 0.049475743 |  | 0.014225 | 0.004023 |
|  | Exact test | 582.7 | 40.15 | 0.049037986 |  | 0.053917 | 0.022639 |
|  | GLM | 592.0 | 41.90 | 0.049785069 |  | 0.062515 | 0.024089 |
|  | DESeq | 400.7 | 8.32 | 0.049205147 |  | 0.004114 | 0.003743 |
|  | Beta t-test | 586.7 | 20.42 | 0.049744667 |  | 0.165699 | 0.010472 |
|  | mBeta t-test | 608.3 | 37.87 | 0.049095016 |  | 0.045627 | 0.02317 |
| Scenario 6 | eBayesian | 887.3 | 28.91 | 0.049840282 |  | 0.034131 | 0.002471 |
|  | Exact test | 901.0 | 12.76 | 0.049361917 |  | 0.048886 | 0.005728 |
|  | GLM | 911.0 | 13.11 | 0.049262212 |  | 0.054955 | 0.007382 |
|  | DESeq | 746.3 | 19.08 | 0.047905044 |  | 0.006171 | 0.005225 |
|  | Beta t-test | 892.0 | 38.22 | 0.0495794 |  | 0.13778 | 0.017347 |
|  | mBeta t-test | 864.3 | 27.06 | 0.04986485 |  | 0.027286 | 0.005041 |
| Scenario 7 | eBayesian | 1880.0 | 37.98 | 0.049924036 |  | 0.016844 | 0.000593 |
|  | Exact test | 1905.0 | 63.26 | 0.049760675 |  | 0.033698 | 0.003521 |
|  | GLM | 1936.3 | 67.33 | 0.04987274 |  | 0.039498 | 0.004252 |
|  | DESeq | 1398.0 | 39.39 | 0.049872216 |  | 0.004724 | 0.002398 |
|  | Beta t-test | 1800.7 | 151.30 | 0.0498517 |  | 0.070921 | 0.011422 |
|  | mBeta t-test | 1887.3 | 74.19 | 0.049352367 |  | 0.018178 | 0.00276 |
| Scenario 8 | ebayesian | 2780.3 | 71.62 | 0.049903333 |  | 0.049308 | 0.00472 |
|  | Exact | 2653.3 | 97.38 | 0.04993212 |  | 0.033475 | 0.008219 |
|  | GLM | 2668.3 | 98.19 | 0.049910444 |  | 0.036769 | 0.008319 |
|  | DESeq | 2275.3 | 81.32 | 0.049506184 |  | 0.004339 | 0.002514 |
|  | Beta t-test | 2509.7 | 40.82 | 0.0498865 |  | 0.060843 | 0.010993 |
|  | mBeta t test | 2599.7 | 29.73 | 0.049559249 |  | 0.014071 | 0.004408 |

Scenario 5: P=10%, Q=30%, A=100, R=3

Scenario 6: P=10%, Q=30%, A=300, R=3

Scenario 7: P=30%, Q=30%, A=100, R=3

Scenario 8: P=30%, Q=30%, A=300, R=3

P = proportion of differentially expressed isoforms.

Q = artificial noise proportion.

A = condition effect.

R = number of replicates.

$\omega=1$ for mBeta t-test method in Scenarios 5-8. Underestimated FDR is indicated in red.

**Table C**: Results of performing statistical methods on simulated data of 18162 isoforms and two conditions in scenarios 9-12, varying proportion and condition effects in the context of five replicates for each condition.

| scenario | Statistical method | number of findings | | estimated FDR |  | true FDR | |
| --- | --- | --- | --- | --- | --- | --- | --- |
|  |  | mean | stdev |  |  | mean | stdev |
| Scenario 9 | eBayesian | 1466.7 | 136.99 | 0.049816998 |  | 0.065909 | 0.017935 |
|  | Exact test | 1455.7 | 135.50 | 0.04942623 |  | 0.075021 | 0.023157 |
|  | GLM | 1492.0 | 157.43 | 0.049120039 |  | 0.092843 | 0.032046 |
|  | DESeq | 1270.7 | 116.81 | 0.04968407 |  | 0.016749 | 0.004976 |
|  | Beta t-test | 1447.7 | 103.58 | 0.049672 |  | 0.116815 | 0.028366 |
|  | mBeta t-test | 1457.3 | 114.02 | 0.048886388 |  | 0.03596 | 0.009967 |
| Scenario 10 | eBayesian | 1822.3 | 95.61 | 0.049864672 |  | 0.104564 | 0.013078 |
|  | Exact test | 1768.3 | 117.50 | 0.049594284 |  | 0.08398 | 0.028075 |
|  | GLM | 1794. 7 | 131.76 | 0.049606781 |  | 0.094059 | 0.033016 |
|  | DESeq | 1593.3 | 125.52 | 0.049260009 |  | 0.021667 | 0.012233 |
|  | Beta t-test | 1772.0 | 67.55 | 0.049848 |  | 0.132159 | 0.025635 |
|  | mBeta t-test | 1697.667 | 67.26 | 0.049301841 |  | 0.032401 | 0.007489 |
| Scenario 11 | eBayesian | 4894. 7 | 289.89 | 0.049930905 |  | 0.081428 | 0.011789 |
|  | Exact test | 4633.3 | 303.32 | 0.049761768 |  | 0.051046 | 0.013995 |
|  | GLM | 4681.3 | 332.24 | 0.049960589 |  | 0.057404 | 0.018121 |
|  | DESeq | 4206.3 | 317.16 | 0.049814792 |  | 0.013886 | 0.005984 |
|  | Beta t-test | 4700. 7 | 362.29 | 0.0493765 |  | 0.070815 | 0.014574 |
|  | mBeta t-test | 4438. 3 | 149.79 | 0.04952921 |  | 0.0124 | 0.000406 |
| Scenario 12 | ebayesian | 5701.0 | 206.72 | 0.049939398 |  | 0.099753 | 0.01504 |
|  | Exact test | 5453. 7 | 100.48 | 0.049737062 |  | 0.048492 | 0.006854 |
|  | GLM | 5454. 7 | 145.40 | 0.049840291 |  | 0.053679 | 0.008784 |
|  | DESeq | 4915.3 | 228.00 | 0.049430541 |  | 0.012464 | 0.005037 |
|  | Beta t-test | 5091. 7 | 128.48 | 0.049957167 |  | 0.066915 | 0.013236 |
|  | mBeta t-test | 5072.3 | 135.57 | 0.049633984 |  | 0.01087 | 0.002098 |

Scenario 9: P=10%, Q=10%, A=100, R=5

Scenario 10: P=10%, Q=10%, A=300, R=5

Scenario 11: P=30%, Q=10%, A=100, R=5

Scenario 12: P=30%, Q=10%, A=300, R=5

P = proportion of differentially expressed isoforms.

Q = artificial noise proportion.

A = condition effect.

R = number of replicates.

$\omega=0.45$for the mBeta t-test method in Scenarios 9-12. Underestimated FDR is indicated in red.
